# The Feeding of Patients and Staff

**Published:** 1916-06-17

**Authors:** 


*To the Editor of* The Hospital.

### The Feeding of Patients and Staff.

*To the Editor of* The Hospital.

Sir,—I notice with much interest, in your issue of June 3, the remarks of "Matron" respecting the food of the patients and staff in institutions for the sick. It is true that most diet tables want overhauling, but in my experience the best and the worst are more honoured in the breach than in the observance. It is time the authorities should realise that paper regulations are of very slight value. The directions of the most skilled dietist would crumble to nothingness when carried out by an incompetent cook, while on the other hand a good cook can render the most unpromising menu palatable and therefore nourishing. It matters little how many ounces of proteid and carbohydrates are swallowed every day, and the best authorities are in dispute as to the requirements of the body. What is more important is the zest with which the food is consumed, the pleasurable anticipation of the diners, and the atmosphere of ease and gaiety which surrounds the meals. By all means let more fruit and vegetables be insisted on, a choice of brown bread be offered, and so on. These things have been advocated for years, and are now the rule rather than the exception. But the cook is the thing, and until institutions pay good wages and set competent women in charge of their kitchens, staff and patients will continue to be ill-fed.—I am, yours, etc.,

Veteran Housekeeper.

